# Providing care to patients with COVID-19 in a reference hospital: health care staff intentional behavior and factors that affect it

**DOI:** 10.3934/publichealth.2021035

**Published:** 2021-05-31

**Authors:** Theodoros Pesiridis, Petros Galanis, Eleni Anagnostopoulou, Athena Kalokerinou, Panayota Sourtzi

**Affiliations:** 1 Public Health Sector, Department of Nursing, National and Kapodistrian University of Athens, Greece; 2 General Hospital of Athens, Athens, Greece

**Keywords:** COVID-19, healthcare staff, behavioral intention, patient care

## Abstract

**Objective:**

The investigation of intentional behavior of hospital staff to care for COVID-19 patients and the study of the factors that influences it.

**Method:**

This is a cross-sectional study, of 261 physicians and nurses working in a COVID-19 reference hospital. Data were collected by an anonymous questionnaire including demographic and professional characteristics and a scale measuring behavioral intention based on the Theory of Planned Behavior of Ajzen. Statistical analysis was performed by SPSS 21.

**Results:**

Mean age of participants was 40.8 years old, while most of them were nurses (75.7%). Behavioral intention mean score was 18.2 (5–21), which shows high intention to care for COVID-19 patients. Bivariate analysis between independent variables showed that behavioral intention mean score was higher for those that had cared for COVID-19 patients and those that did not (19.0% vs. 16.7%, p < 0.001). Multivariate linear regression analysis identified that increased subjective norms (the perceived social pressure to perform or not the behavior) score was associated with increased behavioral intention score (p < 0.001). Also, participants that provided care for COVID-19 patients had higher behavioral intention score (p < 0.001).

**Conclusion:**

Healthcare staff, that cared for COVID-19 patients had high behavioral intention to continue caring for them. This finding could be used to inform policies and training for staff that will be employed in COVID-19 units.

## 1. Introduction

Nurses and physicians are the main health workers that are involved in providing health care in all circumstances and this includes providing care in emergency situations such as, pandemics or disasters. Although historically health care workers have responded to health crises, natural and man-made disasters, little is known about their intentions to care for those who are affected during a pandemic like the one the mankind is going through currently. Alwidyan et al. [1] in a recent study reported that emergency care workers are more inclined to respond to natural disasters than epidemics, although their willingness outweighed the risks for their own health.

Factors that affect the capability of the health care system in general, or a specific hospital, to respond immediately and effectively to crises in order to care for the victims include, among other things, the willingness of health care workers (HCWs) to provide the needed health care, especially when there is a risk to their personal health and safety [2]–[5]. Labraque et al.6 in a literature review about nurses' preparedness for disaster response found that the factors that influence it are previous experience and relevant training. Deynani [7] in another literature review reported also that gender, family situation and access to treatment or availability of preventive measures also affected HCWs willingness to participate in an influenza epidemic.

Behavioral models and theories have been used to investigate the willingness of HCWs to participate in a crisis. The factors that influence HCWs intentional behavior and relate to the decision in participating in the health care of victims can be a valuable guide for those responsible for drawing up action plans as well as for the health structures that will be called to respond to and provide human resources to contribute in this response effectively [8],[9]. One of the models commonly used to study the factors that influence behavioral intention of HCWs is based on Ajzen's theory of planned behavior [10]. Most studies have shown that human behavior is influenced either by intention and perceived control or by one of these two variables alone. In turn, the intention is influenced either by the attitude towards the behavior or by the perceived control of the behavior. Subjective norms that are defined as the perceived social pressure (e.g. from family and friends) to perform (or not) the behavior—usually have the least effect on prediction, but they are considered an indispensable variable of the model [9]–[15].

So far, there are no studies that have examined the intentional behavior of nurses and doctors to participate in the current pandemic. The aim of the present study was to investigate Greek nurses and physicians' intentional behavior for caring for patients with COVID-19 in a tertiary care hospital that became a reference one for the disease following the outbreak in the country and study the factors that affect it.

## 2. Material and methods

### 2.1. Study design

The study was designed as a cross-sectional one and it was performed following the first wave of the pandemic from June to July 2020.

### 2.2. Sample

The sample was a convenience one and was drawn from the total population of physicians and nursing staff (registered nurses and nursing assistants) working in a reference hospital for COVID-19. This hospital is a tertiary public hospital in Athens metropolitan area in Greece, that was made a reference center for the disease immediately after the outbreak—with a capacity of 500 beds and at the time of the study there were 565 nurses and physicians in in full-time employment.

### 2.3. Questionnaire

Data were collected by an anonymous self-reported questionnaire that included demographic and occupational information and a scale measuring intentional behavior. To assess behavioral intent, a structured anonymous questionnaire was developed based on Ajzen's theory [10]. The theory of planned behavior is widely used to predict or explain cognitive and emotional behavior using the relationship of belief-intention-behavior in social psychology [16]. The questionnaire contains 12 phrases answered in a 7-point Likert-type scale divided in four subscales: Behavioral intention (3 phrases and possible score from 3 to 21), Subjective norms (3 phrases and possible score from 3 to 21), Perceived behavioral control (3 phrases and possible score from 3 to 21) and Attitudes (3 phrases and possible score from 3 to 21). The questions included in the scale are provided as supplementary material.

The questionnaire is based on an older version [17] and has been tested for reliability (internal consistency α = 0.57–0.93 and stability r_s_ = 0.54–0.71 p < 0.001), while content validity was checked, and factor analysis was performed. In this study Cronbach's alphas for the Ajzen's Theory of Planned Behavior scales were >0.7 in all subscales indicating good internal consistency of the questionnaire.

### 2.4. Ethical issues

The study protocol was approved by the Ethics Committee of the Department of Nursing of the National and Kapodistrian University of Athens and the Scientific Council of the reference hospital. The questionnaire was accompanied by a letter describing the purpose of the study. Completion of the questionnaire was thought as informed consent to participate in the study. Recipients of the questionnaire were informed that their participation was voluntary, and that the data will be strictly confidential and in accordance with the GDPR rules.

### 2.5. Statistical analysis

Continuous variables are presented as mean, standard deviation, median, minimum value, and maximum value, while categorical variables are presented as numbers and percentages. We used the Kolmogorov-Smirnov test and graphs to test the normality of the distribution of the Ajzen's Theory of Planned Behavior scales. Independent samples t-test, analysis of variance, Pearson's correlation coefficient and Spearman's correlation coefficient were used to estimate differences between independent variables and the behavioral intention score. Then, variables with a p-value less than 0.20 in bivariate analyses were entered into the backward stepwise multivariate linear regression analyses with the behavioral intention score as the dependent variable. Criteria for entry and removal of variables were based on the likelihood ratio test, with entering and remove limits set at p < 0.05 and p > 0.10. We estimated adjusted coefficients beta with 95% confidence intervals and p-values. P-values < 0.05 were considered as statistically significant. Statistical analysis was performed with the Statistical Package for Social Sciences software (IBM Corp. Released 2012. IBM SPSS Statistics for Windows, Version 21.0. Armonk, NY: IBM Corp.).

## 3. Results

### 3.1. Demographic characteristics

Three hundred (300) questionnaires were distributed and 261 (87%) were received completed. Demographic and job characteristics of the participants are shown in Table 1. The questionnaire was complete 63 physicians and 196 nurses. Mean age of the participants was 40.8 years, while most of them were females (76.2%) and married (60.9%). Forty-five-point two percent had dependent children or others, while 27% lived together with vulnerable groups. Regarding job characteristics, 75.7% were nursing staff and 24.3% were physicians, while 34.9% had a MSc/PhD degree. Only 15.8% had attended an educational program on the care of COVID-19 patients and 13.1% had any training that included the acquisition of knowledge and skills on pandemics in the past 2 years. A notable percentage of the participants felt anxiety about pandemics (54.4%).

Descriptive statistics for the Ajzen's Theory of Planned Behavior scales are shown in Table 2. Scores on scales range from 3 to 21 with a mid-point of 12. Mean behavioral intention score, mean subjective norms score, and mean perceived behavioral control score was higher for workers who cared for COVID-19 patients (p < 0.001 in all cases). Mean attitudes score was independent of working status regarding care of COVID-19 patients (p = 0.98).

**Table 1.** Demographic and job characteristics of the participants (N = 261).

Characteristics	Mean/SD	N	%
Gender			
Males		62	23.8
Females		199	76.2
Age	40.8/10.2		
Family status			
Single		78	29.9
Married		159	60.9
Divorced		22	8.4
Widower		2	0.8
Dependent children or others			
Yes		118	45.2
No		143	54.8
Living together with vulnerable persons			
Yes		70	27.0
No		189	73.0
Education level			
University		87	33.3
Technological Institution		108	41.4
Vocational training		66	25.3
MSc/PhD			
Yes		91	34.9
No		170	65.1
Profession			
Physicians		63	24.3
Nursing staff		196	75.7
Work experience (years)	15.0/11.2		
Working department			
Internal medicine		114	43.8
Surgical		21	8.1
Psychiatric		17	6.5
Emergencies		18	6.9
ICU		82	31.5
Outpatients		8	3.1
Educational program on the care of COVID-19 patients			
Yes		41	15.8
No		218	84.2
Participation in care of COVID-19 patients			
Yes		165	66.0
No		85	34.0
Previous training for pandemics			
Yes		34	13.1
No		225	86.9
Anxiety level about pandemics			
None		16	6.1
Low		39	14.9
Moderate		64	24.5
High		91	34.9
Very high		51	19.5

**Table 2.** Descriptive statistics for the Ajzen's Theory of Planned Behavior scales.

Scale	Mean	Standard deviation	P-value^a^
Behavioral intention	18.2	3.4	<0.001
HCWs who have cared for COVID-19 patients	19.0	2.4	
HCWs who have not cared for COVID-19 patients	16.7	4.1	
Subjective norms	17.4	3.6	<0.001
HCWs who have cared for COVID-19 patients	18.2	3.2	
HCWs who have not cared for COVID-19 patients	16.2	3.9	
Perceived behavioral control	18.2	3.4	<0.001
HCWs who have cared for COVID-19 patients	12.9	3.0	
HCWs who have not cared for COVID-19 patients	11.5	3.0	
Attitudes	14.7	0.7	0.98
HCWs who have cared for COVID-19 patients	14.7	0.7	
HCWs who have not cared for COVID-19 patients	14.7	0.7	

Note: ^a^ independent samples t-test.

Bivariate analysis between independent variables (demographic and job characteristics of the participants) and the behavioral intention score are shown in Table 3, while multivariate linear regression analysis with the behavioral intention score as the dependent variable are shown in Table 4. According to bivariate analysis, working department (p = 0.01), participation in the care for COVID-19 patients (p < 0.001), anxiety level about pandemics (p = 0.005), subjective norms (p < 0.001) and perceived behavioral control (p < 0.001) were associated with higher mean of the behavioral intention score. Multivariate linear regression analysis identified that increased subjective norms score was associated with higher mean behavioral intention score (p < 0.001). Also, participants that provided care for COVID-19 patients had higher mean behavioral intention score (p < 0.001).

**Table 3.** Bivariate analysis between independent variables and the behavioral intention score.

Independent variables	Behavioral intention score		
	Mean	SD	P-value
Gender			0.18^a^
Males	18.6	2.7	
Females	18.0	3.6	
Age		−0.09^b^	0.17^b^
Family status			0.71^a^
Singles/widowers/divorced	18.1	3.2	
Married	18.2	3.5	
Dependent children or others			0.82^a^
Yes	18.1	3.8	
No	18.2	2.9	
Living together with vulnerable persons			0.1^a^
Yes	17.6	4.0	
No	18.4	3.0	
Education level			0.4^c^
University	18.5	2.9	
Technological Institution	17.9	3.5	
Vocational training	18.0	3.7	
Profession			0.59^a^
Physicians	18.3	2.8	
Nurses	18.1	3.6	
Work experience (years)		−0.04^d^	0.53^d^
Working department			0.01^c^
Internal medicine	17.7	3.5	
Surgical	18.4	2.4	
Psychiatric	16.1	4.6	
Emergencies	18.0	3.8	
ICU	19.0	2.9	
Outpatients	19.0	2.3	
Educational program about care for COVID-19 patients			0.14^a^
Yes	18.9	2.5	
No	18.1	3.4	
Participation in care for COVID-19 patients			<0.001^a^
Yes	19.0	2.4	
No	16.7	4.1	
Previous training for pandemics during the last two years			0.15^a^
Yes	19.0	2.3	
No	18.1	3.4	
Anxiety level about pandemics		−0.17^d^	0.005^d^
Subjective norms		0.67^b^	<0.001^b^
Perceived behavioral control		0.31^b^	<0.001^b^
Attitudes		0.12^b^	0.06^b^

Note: SD: standard deviation; ^a^ independent samples t-test; ^b^ Pearson's correlation coefficient; ^c^ analysis of variance; ^d^ Spearman's correlation coefficient.

**Table 4.** Multivariate linear regression analysis with the behavioral intention score as the dependent variable.

Independent variables	Coefficient beta	95% confidence interval for beta	P-value	R^2^
Subjective norms	0.56	0.47 to 0.65	<0.001	45%
Participation in care for COVID-19 patients vs. nonparticipation	1.29	0.60 to 1.98	<0.001	

## 4. Discussion

This study investigated Greek nurses and physicians' intentional behavior for caring for patients with COVID-19 in a reference hospital and studied the factors that affect it. Demographic and job characteristics of the participants are comparable to another study performed recently in Greece [18]. So far, no other study has been published on the intentional behavior of hospital staff to care for patients with COVID-19, therefore findings are compared with previous published work on similar situations. The main finding of this study is that HCWs that had cared for COVID-19 patients during the first wave of the pandemic showed higher intentional behavior compared to those that did not have that experience.

Although the study was performed after the first wave of the pandemic in a reference hospital, the proportion of participants who reported that they had attended an educational program about the care of COVID-19 patients or for pandemics in general was small. This could be explained by the inadequate preparedness of the Greek Health System for such a public health problem. It also shows that lessons taught by previous epidemics or other public health threats have not been learned. It is therefore, necessary for health services to question existing policies and available training of HCWs and take all necessary measures to plan, implement and evaluate the needed changes in order to prepare HCWs to be ready, willing, confident and safe when they are asked to provide care in such demanding and risky situations as other studies during previous epidemics. such the H1N1 have shown [5],[17],[19].

A notable percentage (54.4%) of the participants felt anxiety about pandemics. Such a finding that is not surprising considering the limited knowledge about the Severe Acute Respiratory Syndrome Coronavirus 2 (SARS-CoV-2) HCWs had available during the first wave of the pandemic and at the time this study was performed. This anxiety could be due to the perceived personal risk by the pandemic, as well as because of the limited ability to face and or solve the patient's problems, but it did not prevent them from participating in combating it. This finding is compatible with that reported by Alwidyan et al. [1] that emergency care workers although willing to participate in the care of victims of a crisis showed more concern when invited to care of disease outbreak than a natural disaster.

Descriptive statistics for the Ajzen's Theory of Planned Behavior subscales showed that mean values in all were above the mid-point; that means that participants were positive in caring for COVID-19 patients. This finding is in accordance with that reported by Ko et al. [11] in the case of SARS epidemic, and shows that HCWs are true to their call of duty, that is to care for those in need regardless of the situation.

Demographic and job characteristics of the participants that were found to influence intentional behavior score were the hospital department they were working and actual participation in the care for COVID-19 patients. Regarding the hospital department that participants worked and had higher behavioral intention was ICU and outpatients; in both with more contact with COVID-19 patients, a finding that relates to the higher intention of participants that had already cared for COVID-19 patients. It is of importance to refer that in other studies, gender, family situation—being married, having children or other persons in need for care—level of education and experience seem to influence behavioral intention to care for patients/victims or disease outbreak, something that was not supported by our findings [6]–[8],[11],[20]–[23]. This could be due to the nature of the pandemic and the disease itself, something that the healthcare staff had not encountered before. Healthcare staff during the COVID-19 pandemic had to deal with an unpredictable disease, while the knowledge level was very limited especially in the first waves of the pandemic. It is reasonable that demographic characteristics in our study have not played a significant role since subjective norms and participation in care for COVID-19 patients were very strong predictors of intentional behavior mitigating the action of other possible determinants. There is a need for studies with larger samples to clarify the role of demographic characteristics with regards to intentional behavior.

The level of anxiety about pandemics HCWs experienced, subjective norms and perceived behavioral control were associated with intentional behavior score. Participants in the study by Alwidyan et al. [1] although expressed varying concerns about working during disasters and pandemic conditions, everyone felt willing and obliged to go to work despite the perceived high risk for some of them to work in some conditions. Subjective norms and perceived behavioral control were significantly correlated with behavioral intention, while attitudes had a borderline correlation (p = 0.06). Ko et al. [11] and Ejeta et al. [9] also reported similar findings. In our study, subjective norms include family/friends opinion about HCWs' decision to provide care to patients with COVID-19, and nurses' obligation to care for these patients. Nurses with a family/friend environment that supports care to COVID-19 patients feel more confident to provide this care. Additionally, HCWs that believe it is their duty to take care of COVID-19 patients have a more positive behavior and attitude with regards to these patients. Also, we found that increased perceived behavioral control was related with increased behavioral intention. Perceived behavioral control refers to the ability that HCWs had to decide themselves to provide care to COVID-19 patients. Moreover, healthcare provision to these patients fulfills HCWs' professional role giving them the sense that take control of their behavior.

Multivariate linear regression analysis identified that increased subjective norms score was associated with increased behavioral intention score (p < 0.001). Also, participants that provided care for COVID-19 patients had higher behavioral intention score (p < 0.001). This finding is in accordance with the study by Oh et al. [22], who in their study during the Middle East Respiratory Syndrome (MERS) 2015 outbreak found that nurses who were involved in patient care with the disease had higher intention to care for them.

This study has some limitations due to its design; it was designed as a cross-sectional one, it was performed in one hospital and used a convenience sample. Therefore, the findings cannot be generalized and can be used as a guide for future studies as well as for organizing relevant education for health care staff.

## 5. Conclusions

The present study found that physicians and nurses are willing to provide care to patients with severe disease such as COVID-19 and this is more apparent for those who had already worked with such patients, irrespective of having already attended specific education. Education on the problem was found inadequate and although this could be justified due to the abrupt appearance of the problem, there is sufficient evidence from previous epidemics to be used as basis for designing and implementing appropriate educational programs, especially for lifelong learning. It is also necessary the findings of the present study to be used to inform future policies on responding to health care crises such as this pandemic.
